# Supplementary material for: The Systems Biology Research Tool: evolvable open-source software
Source: BMC Syst Biol. 2008 Jun 29;2:55. doi: 10.1186/1752-0509-2-55 (PMC2446383; doi:10.1186/1752-0509-2-55)
Supplement: Additional file 1 — SBRT Archive. An archive of the current version of the Systems Biology Research Tool. [file 1752-0509-2-55-S1.zip › sbrt-1.4.0/doc/developers_guide/api/sbrt/shell/proc/fba/SimpleRxnFileTranslator.html]

SimpleRxnFileTranslator


|  |  |  |
| --- | --- | --- |
| **Overview** \| **Package** \| **Class** \| **Use** \| **Tree** \| **Deprecated** \| **Index** \| **Help** | |  |
| **PREV CLASS**   NEXT CLASS | **FRAMES**    **NO FRAMES**     **All Classes** |  |
| SUMMARY: NESTED \| FIELD \| CONSTR \| METHOD | DETAIL: FIELD \| CONSTR \| METHOD |  |


---


## sbrt.shell.proc.fba Class SimpleRxnFileTranslator

```
java.lang.Object
  sbrt.shell.proc.fba.SimpleRxnFileTranslator
```

---

``` public class SimpleRxnFileTranslator extends java.lang.Object ```

This class is used to translate simple reaction files from, say,
spreadsheets into FBA Reaction Files.

**Author:**
:   This class was written and documented by Jeremiah Wright while in the
    Wagner lab.

---

**Constructor Summary**

`SimpleRxnFileTranslator()`
  
          Constructs a new translator.


| **Method Summary** | |
| --- | --- |
| `IrrevRxnFormatV2` | `getInputIrrevRxnFormat()`             Returns the parser used for irreversible reactions in the input file. |
| `RevRxnFormatV2` | `getInputRevRxnFormat()`             Returns the parser used for reversible reactions in the input file. |
| `EqualsMapEntryFormatV1` | `getLineFormat()`             Returns the format used for individual lines in the input and output files. |
| `IrrevRxnFormatV1` | `getOutputIrrevRxnFormat()`             Returns the formatter used for irreversible reactions in the output file. |
| `void` | `translate(java.lang.String simpleRxnFileName, java.lang.String outputFileName)`             Translates a simple reaction file into an FBA Reaction File. |
| `void` | `translate(java.lang.String simpleRxnFileName, java.lang.String outputFileName, java.io.PrintWriter out)`             Translates a simple reaction file into an FBA Reaction File. |

**Methods inherited from class java.lang.Object**

`clone, equals, finalize, getClass, hashCode, notify, notifyAll, toString, wait, wait, wait`

**Constructor Detail**

### SimpleRxnFileTranslator

```
public SimpleRxnFileTranslator()
```

:   Constructs a new translator.


**Method Detail**

### getInputRevRxnFormat

```
public RevRxnFormatV2 getInputRevRxnFormat()
```

:   Returns the parser used for reversible reactions in the
    input file.

    :   **Returns:**: the parser used for reversible reactions in the provided input file.

---


### getInputIrrevRxnFormat

```
public IrrevRxnFormatV2 getInputIrrevRxnFormat()
```

:   Returns the parser used for irreversible reactions in the
    input file.

    :   **Returns:**: the parser used for irreversible reactions in the provided input file.

---


### getOutputIrrevRxnFormat

```
public IrrevRxnFormatV1 getOutputIrrevRxnFormat()
```

:   Returns the formatter used for irreversible reactions in the
    output file.

    :   **Returns:**: the formatter used for irreversible reactions in the output file.

---


### getLineFormat

```
public EqualsMapEntryFormatV1 getLineFormat()
```

:   Returns the format used for individual lines in the input and
    output files.

    :   **Returns:**: the format used for individual lines in the input and output files.

---


### translate

```
public void translate(java.lang.String simpleRxnFileName,
                      java.lang.String outputFileName)
```

:   Translates a simple reaction file into an FBA Reaction File.

    :   **Parameters:**: `simpleRxnFileName` - the name of the simple reaction file.: `outputFileName` - the desired name of the FBA Reaction File.

---


### translate

```
public void translate(java.lang.String simpleRxnFileName,
                      java.lang.String outputFileName,
                      java.io.PrintWriter out)
```

:   Translates a simple reaction file into an FBA Reaction File.

    :   **Parameters:**: `simpleRxnFileName` - the name of the simple reaction file.: `outputFileName` - the desired name of the FBA Reaction File.: `out` - the print writer to which descriptive messages will be written.


---


|  |  |  |
| --- | --- | --- |
| **Overview** \| **Package** \| **Class** \| **Use** \| **Tree** \| **Deprecated** \| **Index** \| **Help** | |  |
| **PREV CLASS**   NEXT CLASS | **FRAMES**    **NO FRAMES**     **All Classes** |  |
| SUMMARY: NESTED \| FIELD \| CONSTR \| METHOD | DETAIL: FIELD \| CONSTR \| METHOD |  |


---
